# Promising patient experiences with a smartphone app and remote coaching for improving physical activity and protein intake to enhance recovery after oncological surgery: a multi-methods study

**DOI:** 10.1007/s00520-025-09641-0

**Published:** 2025-06-19

**Authors:** C. van Westerhuis, M. E. de Leeuwerk, E. W. J. van der Werf, H. M. Kruizenga, V. de Groot, E. Geleijn, M. van der Leeden, M. van der Schaaf

**Affiliations:** 1 School of Physiotherapy, Faculty of Health, Amsterdam University of Applied Sciences, Amsterdam, The Netherlands; 2 Department of Rehabilitation Medicine, Amsterdam UMC Location Vrije Universiteit Amsterdam, De Boelelaan 1117, Amsterdam, The Netherlands; 3 Department of Nutrition & Dietetics, UMC Location Vrije Universiteit Amsterdam, De Boelelaan 1117, Amsterdam, The Netherlands; 4 Amsterdam Movement Sciences, Ageing & Vitality, Amsterdam, The Netherlands; 5 Amsterdam Gastroenterology Endocrinology Metabolism, Amsterdam, The Netherlands; 6 Faculty of Health, Center of Expertise Urban Vitality, Amsterdam University of Applied Sciences, Amsterdam, the Netherlands; 7 Department of Rehabilitation Medicine, Amsterdam UMC Location University of Amsterdam, Meibergdreef 9, Amsterdam, The Netherlands

**Keywords:** Cancer, Surgery, Supportive Care, Telerehabilitation

## Abstract

**Purpose:**

The Optimal Physical Recovery After Hospitalization (OPRAH) Intervention is a blended intervention, combining a smartphone app for self-monitoring of physical activity and protein intake with coaching from a physiotherapist and dietician, to enhance the functional recovery after gastro-intestinal or lung cancer surgery. The aim was to evaluate patients’ experiences with the OPRAH intervention.

**Methods:**

This study used a multi-method approach, incorporating semi-structured interviews and an online questionnaire. The questionnaire included the System Usability Scale (SUS) to evaluate the smartphone app. Sixty-eight patients were invited to complete the online survey, 58 providing responses. Purposeful sampling guided the selection of participants for interviews (*n* = 24), which focused on experiences with the application, activity tracker, and remote coaching. The interview data were analyzed qualitatively using an inductive thematic approach.

**Results:**

The app was rated with an excellent usability (mean ± SD SUS of 86.2 ± 12.0) and 97% of the patients would recommend the intervention to others. Patients reported that the intervention enhanced their understanding of their recovery process, motivated them to achieve protein and physical activity goals, and contributed to their overall recovery. The combination of app usage and monitoring by a physiotherapist and dietician fostered a sense of being supported. However, patients suggested that the intervention could be improved by further tailoring it to individual needs, detailing the nutritional component of the app, and redesigning the activity tracker bracelet.

**Conclusions:**

The OPRAH intervention demonstrated excellent system usability and predominantly positive experiences. Incorporating patient recommendations could further support its integration into regular healthcare.

**Supplementary Information:**

The online version contains supplementary material available at 10.1007/s00520-025-09641-0.

## Background

With increasing oncology diagnoses and improved curative treatments, the number of cancer survivors is rising, particularly for patients with lung and gastrointestinal (GI) malignancies [1]. Surgery often provides the best medical outcome for these patients, but frequently results in impaired physical recovery [2, 3]. The body’s stress response to surgery accelerates catabolism, causing muscle mass loss and reduced physical functioning. This contributes to complications, increased morbidity and mortality, and diminished quality of life [2–8]. Muscle mass loss is mainly attributed to reduced muscle sensitivity to the anabolic effects of protein intake after surgery and reduced physical activity [3, 7–9]. Therefore, adequate physical activity and nutrition are essential for preventing further muscle mass loss and improving one-year postoperative survival rates [10–14]. Together, these factors synergistically help maintain and rebuild muscle mass, supporting better recovery in physical functioning after surgery [15–17].

While the importance of adequate physical activity and protein intake levels to reduce loss in muscle mass and physical functioning after lung and gastrointestinal cancer surgery is well recognized, many patients struggle to achieve these goals [16–18]. Consequently, support from a physical therapist and dietitian is important during the recovery process. However, healthcare providers face challenges in delivering consistent support due to constraints in time, work structure, finances, and staffing [19]. As a result, patients are often expected to take more responsibility for their own recovery, making self-management essential [20]. While apps and activity trackers can help improving physical activity and nutritional behaviors, their effectiveness increases when combined with professional coaching [21]. Blended care, combining technology and remote coaching, has shown promise in delivering personalized and efficient care. Studies indicate that eHealth can enhance postoperative recovery in oncology patients by promoting self-management, increasing motivation and providing more personalized care and data-driven feedback, which may ultimately improve patient outcomes [22–24]. However, no intervention has been developed to support post-discharge recovery in patients following gastro-intestinal or lung cancer surgery that focuses on both physical activity and protein intake.

Therefore, to support patients in achieving adequate physical activity and protein intake after oncological surgery, we developed the Optimal Physical Recovery After Hospitalization (OPRAH) intervention. The Behavioral Change Wheel was used to develop the intervention, identifying which behavioral change techniques could increase patients’ ability, opportunity and motivation to improve their protein intake and physical activity [25]. This resulted in an intervention with the aim of promoting self-management during recovery through a smartphone application paired with an activity tracker, supported by remote coaching from a physiotherapist and dietitian. This approach also enables healthcare professionals to monitor patients, provide timely interventions, and better coordinate care to optimize recovery.

Patients’ subjective experiences can provide valuable contextual insights, facilitating the evaluation of whether and how the intervention enhances the desired behavior within this patient group. These insights can generate valuable knowledge for advancing the implementation of scientific evidence into practice and contribute to future research projects. Therefore, the aim of this study is to evaluate patients’ experiences with the OPRAH intervention, focusing on its practical application and implementation into rehabilitation programs.

## Methods

### Design

We conducted a multi-method study, combining quantitative surveys with in-depth semi-structured interviews using inductive thematic analysis as described by Braun and Clarke [26]. This study was part of the Dutch multicenter single-blinded OPRAH randomized controlled trial (RCT) at Amsterdam UMC and St. Antonius Nieuwegein, the study protocol of which has been previously[27]. The study protocol was approved by the Medical Ethical Research Committee (METC) of Amsterdam UMC, location VUmc (METC 2021.0627).

### Eligibility criteria and recruitment

Patients scheduled for curative intent surgery for gastrointestinal cancer, including esophageal and stomach cancer (upper GI), colorectal and hepato-pancreato-biliary (HPB) cancer, or lung cancer with a planned hospital stay of ≥ 2 nights, aged 18 years or older, and able to fill in online questionnaires in Dutch, were eligible for the OPRAH study. Exclusion criteria were pulmonary wedge resection, surgery with open/close procedure, having no access to a mobile device compatible for applications, less than 5 days between inclusion and surgery, wheelchair dependence, a Mini-Mental State Examination (MMSE) ≤ 24 and already participating in a conflicting study. Patients were recruited from two hospitals in the Netherlands: Amsterdam UMC, location VUmc and St. Antonius, location Nieuwegein [27]. At one week after the end of the intervention period, patients in the intervention group received a questionnaire focused on their recovery and experiences of the intervention. The patients received an email to complete the online survey on a secured web-based system (Online PROMS). Additionally, patients were approached for an in-depth interview. A purposive sampling approach was applied to assemble a heterogeneous participant sample regarding diversity in age, gender, and kind of surgery to enhance transferability of the findings. No exclusion criteria were used. Participation was voluntary.

### OPRAH intervention

**Fig. 1:**
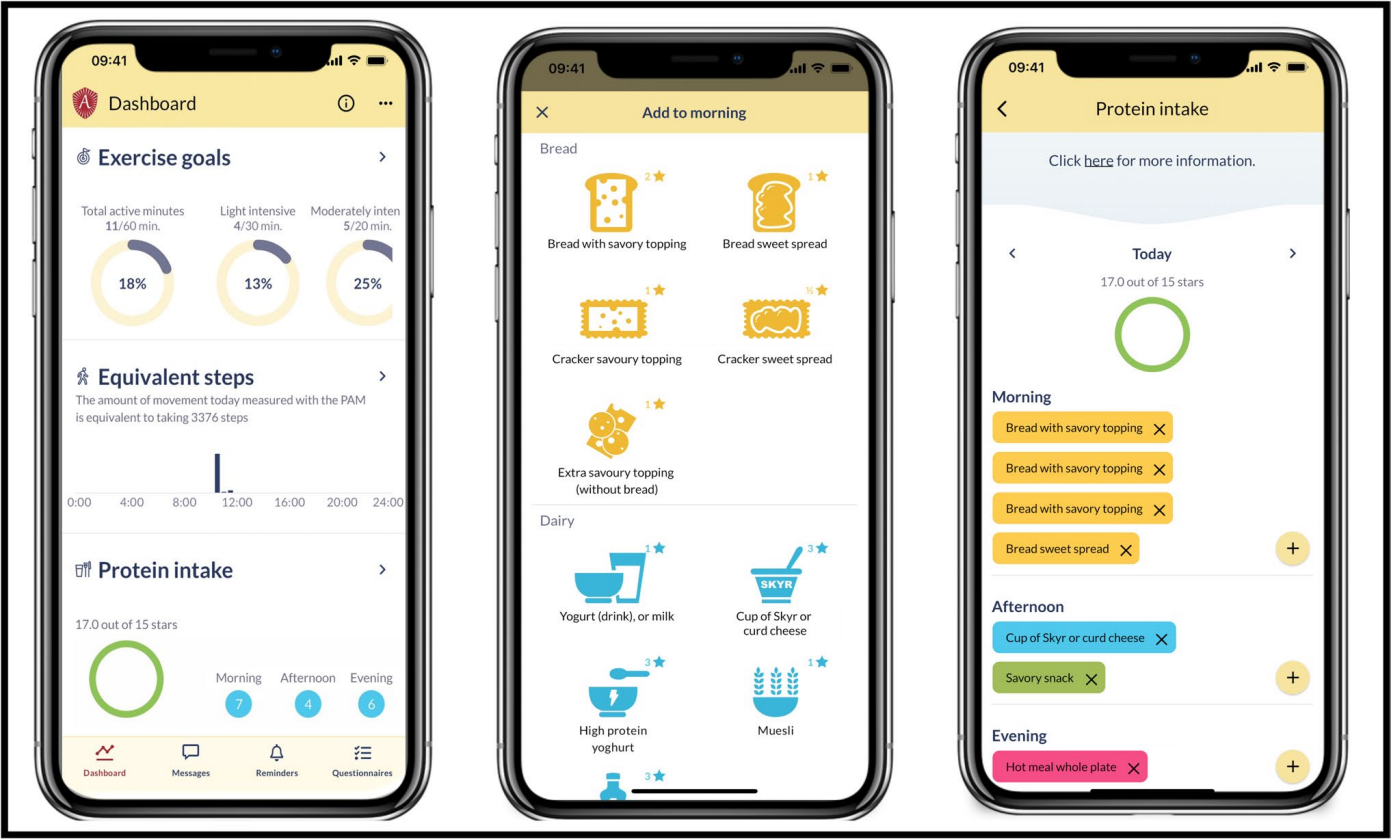
Screenshots of the English language version on the Atris app

The OPRAH intervention was developed with the use of the Behavioral Change Wheel, resulting in 15 Behavioral Change Techniques to improve the patient’ capability, opportunity and motivation to improve their physical activity and reach their protein intake goals after hospital discharge [27]. Using a smartphone app and activity tracker, patients’ self-management is encouraged with the ultimate goal of the intervention a faster and better recovery in physical functioning. Patients in the intervention group received access to the Atris application on their mobile phone and a corresponding activity tracker (PAM) about one week before surgery, both supported by the Atris software (Peercode B.V. Geldermalsen, the Netherlands) and the eiFIT method [28]. The app allowed patients to monitor their physical activity and register their daily protein intake through a simple in-app registration tool combined with personalized goals (See Fig. 1). Patients wore the PAM continuously around their ankle and recorded their protein intake starting five days before surgery. During hospitalization, the physiotherapist and dietician guided patients in using the app. As hospital discharge approached, patients worked with the physiotherapist and dietician using a Shared Decision Making (SDM) process to set goals for physical activity and protein intake after discharge. After leaving the hospital, patients wore the PAM 24 h a day and recorded their protein intake daily for 3 months. They received remote coaching via phone and the application chat function from their physiotherapist and dietician. Patients could ask questions through the app’s chat function, and healthcare providers could send additional information. The primary goal for physical activity was to return to pre-surgery levels, and the goal for protein intake was to meet personal daily requirements. If recovery stagnated and goals were not met, the physiotherapist or dietician could contact the patient to identify potential barriers. Sub-goals and the level of coaching were tailored to individual needs and preferences using the SDM process. Motivational Interviewing (MI) techniques were applied during coaching sessions to support patients'self-management [27].

### Data collection

#### Online questionnaire

The questionnaire consisted of the Dutch translation of the System Usability Scale (SUS) and 16 additional questions about the acceptability, satisfaction and added value of the application. The SUS contains ten statements about efficiency, learnability, and satisfaction and has been validated to assess the usability of electronic systems [29]. Patients indicated the degree of agreement with each statement on a 5-point Likert scale. The total SUS score ranges from 0 to 100, with a score of ≥ 70 considered good [29]. See Supplementary File 1 for the questionnaire.

#### Interviews

Semi-structured interviews were conducted by phone or at the hospital between April 2023 and April 2024 after obtaining written consent. Interviews were scheduled after the 3-month intervention period. They were informed that their data will remain confidential. Interviews were conducted by trained researchers MEdL and CvW. All interviews were audio recorded and transcribed verbatim by CvW and EvdW. The interview guide was based on the OPRAH pilot study (METC 2021.0112) focusing on evaluating patients’ experiences and recommendations across three topics of the OPRAH study: the “Atris app,” “coaching,” and the “duration of the intervention” (Supplementary File 2). Researchers aimed the interview to be between 20 and 40 min. After fifteen interviews, a reflection meeting was held with the research team, MedL, CvW, MvdL, and MvdS, to review the interview guide and interviewer techniques.

#### Descriptive data

Demographic and clinical data were collected retrospectively from electronic medical records. Preoperative physical functioning was measured using the Computer Adaptive Testing Dutch-Flemish Patient Reported Outcome Measure Information System for Physical Functioning (CAT PROMIS PF) questionnaire[30].

### Data analysis

Participants characteristics were described as mean (SD), median (IQR) or percentage. The mean (SD) SUS was calculated using the method described in the study by Brooke [29]. Subgroup analysis based on age, sex, and tumor location were performed to explore potential differences in SUS. Additional questions about user experiences were presented as percentages. SPSS (version 28; IBM Corp) was used for quantitative data analysis of the questionnaire.

The interviews were analyzed using a thematic analysis to investigate patients’ experiences about the OPRAH intervention to identify advantages, disadvantages and recommendations. Three evaluators (MEdL, CvW, and EvdW) followed the six-phase process outlined by Braun and Clark (See Supplementary File 3 for details) [26]. During each phase of the thematic analysis, specific activities were performed to establish trustworthiness. The evaluators started phase one till four with the first six transcripts. In the first phase, the evaluators read and familiarized themselves with qualitative data, while making initial notes about potential themes. After an in-depth reading of the transcripts, during phase two, the three evaluators independently generated initial codes and recognized themes. Periodic meetings with the three analyzing evaluators were held throughout the coding procedure for peer debriefing. During phase three, the evaluators grouped the generated codes, supported by texts, such as words, phrases, statements or entire paragraphs, from which to extract meaningful core themes. This process of inferential analysis was performed independently by the three evaluators. Afterwards, during phase four, potential themes were reviewed during reflexive meetings, to identify patterns of shared meaning underpinned by central ideas (domains). These meetings helped ensure that the scope and focus of each theme were agreed upon, and to minimize personal bias. After finishing phases four for the first six transcripts, the first four phases were repeated for the other transcripts, before continuing to phase five and six. During phase five, themes were revised and defined with the three evaluators together, with the description of a set of codes representing single words or short phrases that best described the experiences of the patients. The results were produced during phase six, categorized by themes and supported by illustrative quotes. All decisions and changes were documented in an audit trail, along with field notes and memos. MAXQDA 2022 was used for the coding process.

### Reflexivity

Credibility of data was achieved by data triangulation, using both questionnaire data and interview paraphrasing. The team’s diverse expertise enriched the exploration of patients’ experiences. MEdL, a hospital physiotherapist and PhD candidate, had experiences with quantitative analyses and offered insights from previous pilot studies with similar patients. CvW, with a background in primary care physiotherapy and nutrition, and PhD candidate, had experiences conducting interviews and was trained in qualitative research. In addition to MEdL and CvW, the research team involved in the analysis consisted of individuals with diverse backgrounds, including dietetics and nutrition (HK), physiotherapy (MvdL, MvdS, EG), rehabilitation medicine (VdG) and lifestyle coaching (EvdW). Efforts were made to mitigate potential biases by promoting open discussions and critical reflections within the research team. The COnsolidated criteria for REporting Qualitative research (COREQ) guidelines were followed to ensure rigorous reporting [31].

## Results

A total of 387 patients were assessed for trial eligibility, of which 193 consented to participate in the study. Of the 90 patients assigned to the intervention group, 13 were excluded before the first follow-up measurement for various reasons, such as withdrawal from participation, cancelled surgery, surgery that deviated from original plan and thus no longer met inclusion criteria, or discharge to a rehabilitations center. Of the 73 patients that started with the intervention, 63 completed the intervention period. Reasons for discontinuation were not interested in continuation (N=3), other medical treatment (N=2), not satisfied with the intervention (N=2), hospital readmission (N=2), and palliative treatment (N=1). 

### SUS and additional questions

The online questionnaire was sent to 68 patients, of which 58 (85%) completed the questionnaire. Patient and perioperative characteristics of the respondents are presented in Table 1. No significant differences were found between the characteristics of the respondents and non-responders (data not shown).

**Table 1:** Patient and perioperative characteristics of the respondents

	**Variable**	**Respondents questionnaire** **(*N* = 58)**	**Respondents Interview** **(*N* = 24)**
**Patient characteristics**	**Sex (male), n (%)**	33 (57%)	13 (54%)
	**Age (years), mean ± SD**	64 ± 10.2	64 ± 10.7
	**BMI, mean ± SD**	26.1 ± 4.9	25.5 ± 4.8
	**Educational Level** Low Middle High	10 (17%) 28 (48%) 20 (35%)	4 (17%) 11 (46%) 9 (37%)
	**Household** Living alone Living together	13 (22%) 45 (78%)	6 (25%) 18 (75%)
	**Tumor location, n (%)**		
	Lung Upper GI HPB Colorectal	18 (31%) 26 (45%) 10 (17%) 4 (7%)	10 (42%) 10 (42%) 3 (13%) 1 (4%)
	**ASA grade ≥ 3, n (%)**	16 (28%)	6 (25%)
	**Charlson Comorbidity Index, mean ± SD**	5.2 ± 2.0	5.3 ± 2.1
	Preoperative physical functioning*, mean ± SD	47.7 ± 5.8	47.5 ± 4.9
**Perioperative characteristics**	**Hospital, n (%)** Amsterdam UMC St. Antonius Nieuwegein	53 (91%) 5 (9%)	22 (92%) 2 (8%)
	**Type of surgery, n (%)** Laparoscopic Open	44 (76%) 14 (24%)	18 (75%) 6 (25%)
	**Comprehensive Complication Index,** **mean ± SD** (if complications = yes)	28.6 ± 9.7	27.1 ± 7.9
	**Length of hospital stay, median (IQR)**	6 (4)	7 (4)
	**Hospital readmission within 30 days after hospital discharge, n (%)**	6 (10%)	2 (8%)
	**Postoperative treatment, n (%)** No treatment Chemo- or immunotherapy Radiation therapy Unknown **Number of days between hospital discharge and first treatment, mean ± SD**	40 (69%) 16 (27%) 1 (2%) 1 (2%) 74 ± 17	20 (83%) 3 (13%) 1 (4%) 0 (0%) 74 ± 16

*Measured with the CAT PROMIS Physical Functioning, ASA = The American Society of Anesthesiologists Classification of physical health; BMI = body mass index; upper GI = upper gastro-intestinal (esophagus and stomach); HPB = hepato-pancreato-biliary; Comprehensive Complication Index: complications occurred within 30 days after surgery or during hospital stay;

The mean SUS was 86.2 (SD 12.0). No significant differences were found in the subgroup analysis. Of all respondents, 97% would recommend the use of the PAM and Atris app to other patients after surgery. Patients rated the general experiences of the Atris app with a mean of 7.7 out of 10 (SD 1.2). Other results of the questionnaire are presented in Table 2.

**Table 2:** Summary of results of SUS and additional questions (*N* = 58)

Criteria	Results
System usability Efficiency Learnability Satisfaction	Mean 88.2 (SD 2.8) Mean 90.2 (SD 1.7) Mean 81.6 (SD 4.8)
Average degree for general experiences with the application (0–10)	Mean 7.7 (SD 1.2)
Average degree for the coaching experiences by the physiotherapist (0–10) Number of contacts with physiotherapist > 1x/week 1-2x/two weeks 1-2x/month < 1-2x/month Never Average degree for the coaching experiences by the dietician (0–10) Number of contacts with dietician > 1x/week 1-2x/two weeks 1-2x/month < 1-2x/month Never	7.9 (SD 1.9) N = 7 (12%) N = 12 (21%) N = 27 (47%) N = 9 (16%) N = 3 (5%) 7.8 (SD 1.4) N = 22 (38%) N = 25 (43%) N = 6 (10%) N = 3 (5%) N = 2 (3%)
Number of app openings > 3x/day 1-3x/day Every other day 1x/week < 1x/week Never	N = 20 (34%) N = 30 (52%) N = 7 (12%) N = 1 (2%) N = 0 (0%) N = 0 (0%)

### Interviews

A total of 27 patients were invited to participate in an interview, of which 24 (89%) were willing to participate. Patient characteristics of the interviewed patients are presented in Table 1. Three main themes emerged from the interviews, focusing on patients’ experiences with the app, activity tracker and remote coaching: 1) the advantages of the OPRAH intervention, 2) the disadvantages of the OPRAH intervention, and 3) recommendations to optimize the OPRAH intervention. The three main themes were described with subthemes and supported by quotes. A complete overview of the results is shown in Table 3.

**Table 3.**
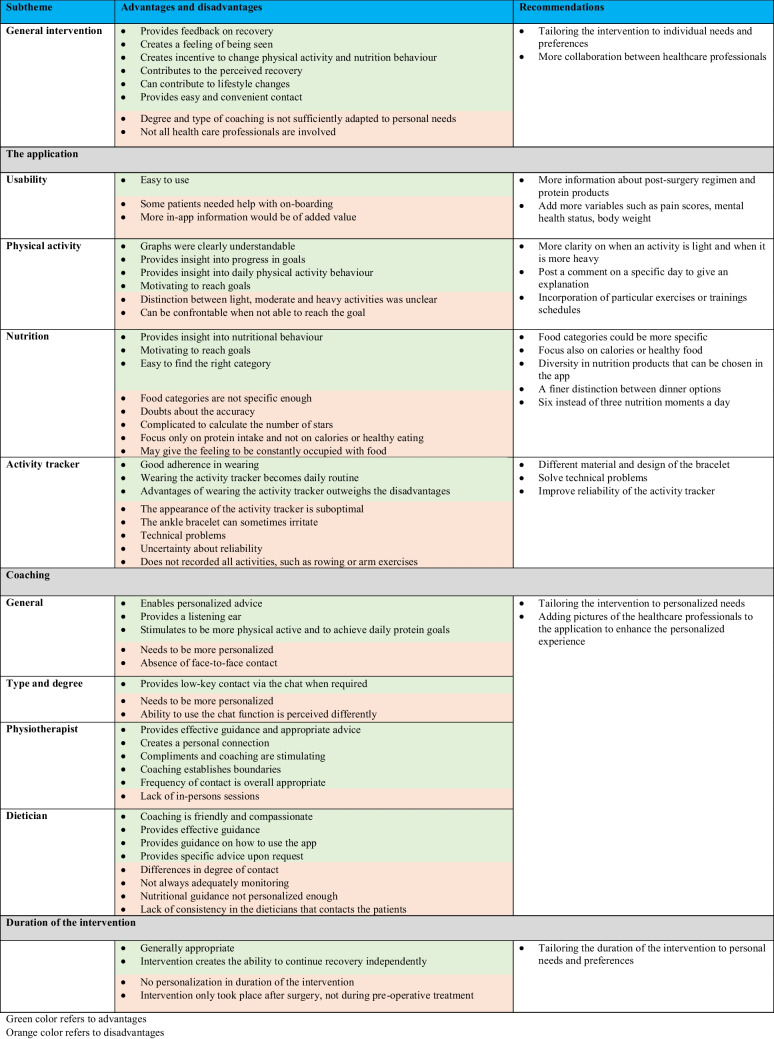
Summary of advantages, disadvantages, and recommendations of the OPRAH intervention

#### Advantages of the OPRAH intervention

Overall, patients had a positive experience with the OPRAH intervention. One of the most frequently mentioned benefits was that patients felt empowered to take an active part in their cancer treatment. Cancer treatment typically felt like a passive period, with patients relying on healthcare professionals for their care (e.g., surgery, radiation, or chemotherapy). The OPRAH intervention gave them the opportunity to take action themselves, through guidance that was personalized to their daily activities, without making it too complex. Not only did it provide a sense of empowerment, but patients also noted that it improved their self-efficacy.

I think I might have encountered more difficulties if I hadn’t been guided in this way. Of course, I’m very stubborn with certain things, so I definitely pushed myself beyond my limits. But this process allowed me to set my boundaries earlier, and that's something I really needed. So, I found that very helpful. [Female, 55 years, HPB-cancer].

The surgery was successful. But you never know what it will be like afterwards. But then along the way you see in the app, ‘hey, I’m hitting my goals’, which in turn gives you more confidence that you can get back to your original level. [Male, 78 years, Upper GI cancer].

In addition to enhancing self-efficacy, patients mentioned that they learned a lot about the importance of sufficient protein intake and regular physical activity. Visualizing their nutrition and physical activity through the app, specific nutritional information, combined with personalized goals, motivated patients to improve their behavior.

I was indeed motivated by the app and the sensor. I was fully aware that it was important to start moving as soon as possible. And to pay attention to my diet, and that for recovery, I needed to eat more proteins. [Female, 58 years, lung cancer].

Almost all patients were convinced that without the OPRAH intervention, and more specific attention to the importance of combining physical activity and protein intake, their recovery would have taken longer. They believed the intervention motivated them to be more physically active at an earlier stage of their recovery. Additionally, some patients mentioned that the intervention also contributed to lifestyle change after the intervention period.

Usually, I do move…. but now I'm extra motivated by the monitor and the sensor. And protein, yes, that’s definitely been a learning moment for me, realizing how important it is for muscle development. So, I’ll definitely take that with me in the future. [Male, 68 years, lung cancer].

The feeling of being seen and supported by the dietician and physiotherapist through the app, was mentioned as a beneficial aspect. This especially helped them in the first weeks after discharge, a period when patients would normally feel quite lonely and insecure. Knowing that healthcare professionals had insight in their recovery in physical activity and protein intake gave patients a sense of safety, and the in-app chat feature made it easy to get in touch if needed.

The contact and the stimulation you get from it. Yes, you have to do it all for yourself, of course. Because that’s what it’s all about, how you do it and not how someone else does it. But it is nice that there is someone watching along and encouraging you. Yes, I don't think this is applicable to everyone, but I think it is for a lot of people. [Female, 69 years, upper GI cancer].

#### Disadvantages of the OPRAH intervention

While patients were generally positive about the OPRAH intervention, they also noted some critical points. One concern involved the digital-only format of the intervention. Some patients would have preferred a combination of face-to face and video consultations with healthcare professionals, while others felt that video consultations would have met their need for more personal contact.

Yes, sometimes I found it frustrating that I felt I couldn't express myself. For example, when I started missing my goals. If you get something like a lung hernia, for instance, you're less able to meet those goals. And no one can see that through the app. [Male, 69 years, lung cancer].

Currently, the intervention focuses solely on tracking daily steps and active minutes to assess physical activity levels. For some patients, this was enough to encourage physical activity and support their recovery. However, patients who recovered more smoothly indicated that they would also benefit from additional advice on how to improve their endurance and strength later in their recovery.

But after just walking, which you naturally do at first, do you also have guidance on what you can do next? Like what kind of exercises? […] That's obviously something that can be incorporated into the app as well, because right now it only showed the minutes of activity. [Female, 58 years, lung cancer].

Another disadvantage mentioned by patients was the design of the activity tracker, which many compared to a prison bracelet. Several patients felt uncomfortable wearing it, as they often received comments from others that reminded them of this association. Some patients found such comments to be annoying, which could potentially have had a demotivating effect on their motivation and behavior.

…And especially since it was during the summer months. For example, at one point I was walking through the garden, and then the neighbor called me, asking when I would be released, you know, that kind of thing. Well, yeah, you know, I found that a bit annoying, but it's just a matter of getting used to it. [female, 67 years, lung cancer].

Specific to the nutrition component of the app, patients felt that the app provided a limited and somewhat inaccurate view of their protein intake. They noted that the app lacked details, making it difficult to accurately record what they had eaten. Additionally, they mentioned that the portion sizes were not always well-suited to this specific patient population, making the nutritional tracking feel less relevant for some. Those components could have influenced the motivating behavior effect of the application on patients.

However, what I did find unfortunate in the app is that you can't specifically check or indicate what proteins you've consumed. So, you have to search for what corresponds to it. And that's quite…yeah, I find that quite a hassle. [female, 64 years, lung cancer].

Because I had esophageal cancer and now have a stomach tube, I can no longer take in more than about 300–350 g or milliliters at a time. Is that half a plate, a quarter of a plate, or a whole plate? I don't know. [male, 64 years, upper GI cancer].

#### Recommendations to optimize the OPRAH intervention

Based on these advantages and disadvantages, patients provided several recommendations to improve the intervention. A more personalized program was suggested, which could take various aspects into account to better suit individual needs. For instance, patients have advised that the duration of the intervention should be tailored, given that recovery is a highly individual process and therefore the length of support required will vary from one patient to another. Also, the frequency of contact between patients and healthcare professionals should be adapted to patients’ needs. In addition, the intervention could be tailored by offering a choice between digital-only, face-to-face, or blended care, designing recovery programs that include endurance and strength training, and allowing the involvement of different healthcare providers, such as dieticians, physiotherapists, doctors, and specialized nurses. Additionally, patients suggested offering the program during both pre- and post-hospitalization phases to maximize benefits.

I don't have a great need for it, but maybe sometimes they should seek more contact. Call more often to check in on how things are going. It went very well for me, but there might be people for whom it's not going as smoothly. Yeah, maybe they should reach out to those people more frequently, I think. [Male, 60 years, upper GI cancer].

Finally, patients strongly recommended redesigning the ankle tracker or selecting a different type of wearable, especially women who wanted to wear dresses or skirts in warmer weather. A more discreet option would make them feel more comfortable, which could positively impact their recovery experience.

Yeah, I've said before, you shouldn't send a black ankle monitor. Send a, what do you call it, a skin-colored ankle monitor. Because when us women walk around with bare legs in the summer, well, it really stands out, I have to say. [Female, 84 years, colorectal cancer].

## Discussion

This study demonstrates the positive patient experiences with the OPRAH intervention and the excellent usability of the app, reflected by a SUS score of 86.2. Patients reported that the intervention enhanced their understanding of their recovery process, motivated them to achieve their protein and physical activity goals, and contributed to their overall recovery and, in some cases, even lifestyle changes. The combination of using an app and being monitored by a physiotherapist and dietician fostered a sense of being supported. However, patients suggested that the intervention could be improved by tailoring it further to individual needs, making the nutritional component of the app more detailed, and redesigning the activity tracker.

The usability of the app has been notably improved compared to our previous studies. The earlier versions of the Atris app reported a mean SUS score of 77.3 (SD 20.7) [32, 33]. The current version scored better in efficiency, learnability, and satisfaction, likely due to pilot study evaluations and app optimization. Enhancements such as alarm or messages for unreached goals, tailored personal goals, optimization of the nutritional part of the app and an extended information folder for application installation and protein-rich products, likely contributed to the improved usability, particularly the learnability, which increased from 74.0 and 76.6 to 90.2. Our systematic review revealed that interventions combining activity trackers with coaching yielded superior outcomes [21]. Consequently, coaching by a physiotherapist and dietician was integrated into the intervention after the first pilot study. The incorporation of coaching may also have enhanced the usability, facilitating low-key contact in case of any uncertainties.

The SUS score of the app is excellent compared to other studies using activity trackers and apps. Jonker et al. reported a mean SUS of 71.3 for a home monitoring system and wrist-worn tracker in older adults’ post-oncological surgery [34]. Low et al. reported a mean SUS of 83.8 for a Fitbit and app in abdominal cancer surgery patients [35]. Van der Linden et al. found similar results with a mean SUS of 85 for colorectal patients using a tracker, app, and food diary perioperatively [36]. The positive feedback observed in these studies aligns with our findings, as patients similarly highlighted the value of gaining insight into their physical activity. Additionally, the studies underscored the importance of providing more personalized care as a key requirement [35].

The qualitative results suggest that the OPRAH intervention addressed the three components of the Behavior Change Wheel: capability, opportunity and motivation [25]. Regarding the capability component of the Behavior Change Wheel, patients mentioned that self-monitoring and goal setting helped them understand how to improve their physical activity and protein intake, which made them capable to improvement and self-management. When facing barriers, they had the possibility to low-key contact with the healthcare professionals to increase their capability. Some patients reported that they found it difficult to discuss these barriers through chat or phone. For these patients, in-person contact might further improve their capability. Regarding opportunity, patients mentioned that the intervention empowered them to act, increasing their sense of control. This was supported by physical opportunities such as in-app prompts to track their progress, as well as social support through access to healthcare professionals via chat. This empowerment may contribute to improved quality of life in patients with cancer [37]. Regarding motivation, patients indicated that the intervention raised awareness about the importance of physical activity and protein intake, which increased their motivation to engage in the desired behavior. Reaching their goals also motivated them to have confidence in being able to recover to pre-operative levels (reflective motivation). Some patients even continued these behaviors after the intervention, suggesting that the intervention also addressed automatic motivation.

An important finding in this study is that despite the personalization already applied in the intervention, patients indicated a need for more personalized care, particularly in the level and amount of coaching. Coaching was added as a component of the intervention, because it facilitated the goal setting and information provision. It was expected that the need for coaching would vary between patients, as the recovery process is likely to differ between patients due to different types of surgery, complications or personal factors. Therefore, the OPRAH intervention did not employ a specific coaching protocol regarding the amount and timing of coaching. It was therefore considered appropriate to provide healthcare professionals the flexibility to match personal needs. This may have led to differences in patients’ experiences with the coaching. Some patients had more or less intensive contact with the physiotherapist or dietician, which was acceptable to them. Some patients reported a need for more frequent contact. Therefore, it is suggested that healthcare professionals should conduct assessment of their patients’ needs before and during the intervention to ensure that these needs are being met. Advising healthcare professionals to identify the patient’s needs could help to optimize the OPRAH intervention, especially in case of remote coaching.

### Strengths and limitations

A strength of this study is the multi-methods approach of interviews and online questionnaires. The utilization of semi-structured interviews enabled a comprehensive exploration of patient experiences and perspectives. The online questionnaire quantitatively measured the usability and experiences of the patients with the intervention. This methodology yielded rich, detailed data, facilitating the identification of nuanced insights into the experiences of the intervention and areas for enhancement.

A limitation of this study is the potential selection bias, as only 17% of the included patients had a low education level, and patients who were unable to complete questionnaires in Dutch were excluded from participation. This limited our ability to explore whether the intervention is usable for this group and meets their needs and preferences, highlighting the importance of addressing this in future research. It should also be noted that patients who consented to participate in studies using apps may be more inclined to perceive the self-monitoring tool as acceptable and beneficial. In our study, this may also have had an impact, as patients open to using apps were more likely to participate. In addition, not all patients were invited for an interview; in some cases, such as recent readmission, patients were not approached in order to avoid overburdening them with study-related tasks. This selective inclusion may have introduced a biased perspective of the overall patient experience, as patients experiencing setbacks or a high level of complaints did not participate in the interviews. Lastly, researchers participated in interventions’ evaluation were involved in the OPRAH study and therefore may have biases about the importance of evaluating patient experiences, and thereby the interpretation of the experiences.

### Practical relevance

The findings of this study highlight the potential for blended interventions, like the OPRAH intervention, in post-operative care. The high usability of the app demonstrates that digital tools can support patient during their recovery by promoting self-management. Combined with personalized coaching from physiotherapists and dieticians, this approach allows for flexible, tailored support that adapts to individual recovery needs. Despite that this intervention was only tested in hospitals, it may also be interesting for the primary care, given the increase in care being moved from hospital towards primary care. However, the need for further personalization, especially in coaching intensity and app features, is key to optimize the intervention.

## Conclusion

The results of our study showed an excellent system usability of the app and predominantly positive user experiences in patients who received the OPRAH intervention after oncological surgery. The results of the interviews indicate that the intervention had a positive impact on the patients’ capacity, opportunity and motivation to engage in physical activity and increase their protein intake following hospital discharge. Patients highly valued this type of support, as the combination with remote coaching provided a low-key communication possibility with involved healthcare professionals. However, patients with lower digital literacy or more complex disease trajectories may have different needs, such as face-to-face support or more individualized goal setting. While the OPRAH intervention appears suitable for integration into routine care, its implementation should be tailored to patient-specific characteristics and needs to ensure broader applicability.

## Supplementary Information

Below is the link to the electronic supplementary material.

Supplementary file1 (DOCX 20 KB)

Supplementary file2 (DOCX 19 KB)

Supplementary file3 (DOCX 15 KB)

## Data Availability

No datasets were generated or analysed during the current study.

## Abbreviations

CAT PROMIS PF: Computer Adaptive Testing Dutch-Flemish Patient Reported Outcome Measure Information System for Physical Functioning
COREQ: COnsolidated criteria for REporting Qualitative research
GI: Gastrointestinal
HPB: Hepato-Pancreato-Biliary
METC: Medical Ethical Research Committee
MI: Motivational Interviewing
MMSE: Mini-Mental State Examination
OPRAH: Optimal Physical Recovery After Hospitalization
PAM: Physical Activity Monitor
RCT: Randomized Controlled Trial
RCTs: Randomizes Controlled Trials
SMD: Shared Decision Making
SUS: System Usability Scale
