# Supplementary material for: Promising patient experiences with a smartphone app and remote coaching for improving physical activity and protein intake to enhance recovery after oncological surgery: a multi-methods study
Source: Support Care Cancer. 2025 Jun 19;33(7):597. doi: 10.1007/s00520-025-09641-0 (PMC12178970; doi:10.1007/s00520-025-09641-0)
Supplement: Supplementary file 1 — Supplementary file1 (DOCX 20 KB) [file 520_2025_9641_MOESM1_ESM.docx]

## Appendix 1

**Evaluation OPRAH study & Atris app**

**General introduction**

Over the past three months you have participated in the OPRAH study. We would like to ask you a number of questions about your experiences.

1. How far have you recovered from your operation?

1 2 3 4 5 6 7 8 9 10

- - 1 = not recovered at all
  - 10 = fully recovered

1. How would you rate the guidance you received from healthcare providers during your recovery after the operation?

1 2 3 4 5 6 7 8 9 10

1. In which group did you participate in the OPRAH study?

- Intervention group / app group
- Control group

**You only need to complete the following questions if you have received the Atris app.**

We would like to ask you a number of questions about your experiences.

The statements below are about the use of the Atris app. Please indicate the extent to which you agree with each statement: 1 = completely disagree, 5 = completely agree. Or give a rating from 1 – 10.

1. How would you rate the Atris app overall?

1 2 3 4 5 6 7 8 9 10

1. I think I would use the Atris app again if I had to have surgery again.
   - 1 = strongly disagree, 2 = disagree, 3 = neutral, 4 = agree, 5 = strongly agree.
2. I find the Atris app unnecessarily complicated.
   - 1 = completely disagree, 5 = completely agree.
3. I find the Atris app easy to use.
   - 1 = completely disagree, 5 = completely agree.
4. I needed help from a technical person to use the Atris app.
   - 1 = completely disagree, 5 = completely agree.
5. I think the various functions of the Atris app are well integrated.
   - 1 = completely disagree, 5 = completely agree.
6. I think there are too many contradictions in the Atris app.
   - 1 = completely disagree, 5 = completely agree.
7. I imagine that most people will quickly learn how to use the Atris app.
   - 1 = completely disagree, 5 = completely agree.
8. I find the Atris app difficult to use.
   - 1 = completely disagree, 5 = completely agree.
9. I felt very comfortable using the Atris app.
   - 1 = completely disagree, 5 = completely agree.
10. I still had to learn a lot of things before I could get started with the Atris app.
    - 1 = completely disagree, 5 = completely agree.
11. I was well guided in learning to use the app.
    - 1 = completely disagree, 5 = completely agree.
12. Using the Atris app has encouraged me to consume more protein.
    - 1 = completely disagree, 5 = completely agree.
13. Using the Atris app has motivated me to exercise more.
    - 1 = completely disagree, 5 = completely agree.
14. Using the Atris app has contributed to my physical recovery.
    - 1 = completely disagree, 5 = completely agree.
15. Using the Atris app was an added value to my rehabilitation process.
    - 1 = completely disagree, 5 = completely agree.
16. Using the Atris app has made me more aware of my protein intake.
    - 1 = completely disagree, 5 = completely agree.
17. Using the Atris app has made me more aware of my exercise behavior.
    - 1 = completely disagree, 5 = completely agree.

We have asked you to wear a PAM motion sensor on your ankle and to keep track of your protein intake in the app. It may happen that it has not always been possible to do this.

1. If you have not always worn the PAM, what was the reason for this?
   - Does not apply
   - I forgot
   - The PAM was not comfortable
   - There were technical problems with the PAM
   - Otherwise, namely: ….
2. If you have not always kept track of your protein intake in the app, what was the reason for this?
   - Does not apply
   - I forgot
   - I couldn't find the right product
   - I thought it was too complicated
   - It takes too much time for me
   - Otherwise, namely: ….
3. On average, how often did you look at the Atris app to view your exercise activity and protein intake?
   - More than 3 times a day
   - 1 -3 times a day
   - Every other day
   - 1x per week
   - Less often than once a week
   - Never
4. How often have you had contact with the dietician of the OPRAH study?
   - > 1x per week
   - 1-2x every two weeks
   - 1-2x per month
   - Less than 1-2x per month
   - Never
5. How would you rate the general guidance provided by the dietitian in the OPRAH study?

1 2 3 4 5 6 7 8 9 10

1. How often have you had contact with the physiotherapist of the OPRAH study?
   - > 1x per week
   - 1-2x every two weeks
   - 1-2x per month
   - Less than 1-2x per month
   - Never
2. How would you rate the general guidance of the physiotherapist of the OPRAH study?

1 2 3 4 5 6 7 8 9 10

1. Would you recommend other patients to use the Atris app if they need surgery?
   - Yes
   - No

Do you have any comments or tips regarding your participation in the OPRAH study?
