# Supplementary material for: Promising patient experiences with a smartphone app and remote coaching for improving physical activity and protein intake to enhance recovery after oncological surgery: a multi-methods study
Source: Support Care Cancer. 2025 Jun 19;33(7):597. doi: 10.1007/s00520-025-09641-0 (PMC12178970; doi:10.1007/s00520-025-09641-0)
Supplement: Supplementary file 2 — Supplementary file2 (DOCX 19 KB) [file 520_2025_9641_MOESM2_ESM.docx]

## Appendix 2

**Main question** : How do patients experience the technology of the OPRAH recovery program, including the in-app triggers and remote feedback, and coaching by a physiotherapist and dietician?

| **Semi-structured interview guide - patient** |  | | |
| --- | --- | --- | --- |
| **Welcome** | **Thank you for participating**  Thank you for participating in this interview, in which I would like to discuss your experience with the OPRAH intervention. My name is Charlotte van Westerhuis and I work as a junior researcher at the OPRAH project. During this interview I would like to discuss your experience with the OPRAH intervention during the three months after your surgery. The aim is that we can optimize the OPRAH intervention, so that hopefully many more people can benefit from this guidance.  You have already signed the informed consent prior to this interview. As previously communicated with you, this interview will be recorded so that we can process it later. The recording will be used exclusively for this research and will be processed completely anonymously. Afterwards you will receive a transcript of this interview. The recording will now start. | | |
| **Introduction** | About … months ago you had abdominal surgery. During your rehabilitation you used the OPRAH intervention, consisting of the Atris app, a movement sensor and remote coaching from a physiotherapist and dietician. During this interview we will discuss nutrition, exercise and recovery. I am also curious about your experience of OPRAH intervention. A distinction will therefore be made between the Atris app, the motion sensor and remote guidance. The interview will last approximately 20 minutes.  Are there any questions at this time? | | |
| **Topic** | **Main question** | **Subtopics** | **Notes** |
| **Introductory question** | How do you think you can influence your recovery?  How could diet and exercise influence this?  Did the OPRAH guidance meet your expectations? |  |  |
| **Preparatory phase**  Introduction to the Atris app | What was your experience during the preparatory period (days before the OR) with getting acquainted with the Atris app? | - First impression Atris app - Information/help |  |
| **Intervention phase**  App  Remote coaching | How were the goals for nutrition and exercise established? And to what extent did you feel that you had a say in this?  What was your experience with the Atris app during the intervention period?  What was it like for you to have to check and enter data in the app yourself?  What was it like for you to gain insight into your diet and exercise? And what did you do with the information about nutrition and exercise that you could view in the app?  In addition to your exercise minutes and protein intake, would you like to get more information from the Atris app?  How did you experience the guidance from the physiotherapist and dietician during your rehabilitation? How did you most prefer to be approached and how often was this?  What differences and/or similarities have you noticed in guidance from the physiotherapist and dietician ?  To what extent did the app's triggers/reminders cause you to take action? | - Feasibility (How feasible were the goals for you?) - Dialogue - Protein intake input - Wearing and connecting the PAM sensor - Any obstacles - By phone - Through the application - Cooperation |  |
| **Review**  General experience Atris app  Added values to patient rehabilitation?  Additions & future | How do you look back on the rehabilitation after your operation?  How did the OPRAH intervention help you during your rehabilitation? What is the core?  Did you feel like you had recovered after 3 months?  How could the OPRAH intervention be improved so that more people can use it in the future?  Should more healthcare professionals be involved during the guidance in the app? If so, which one and how would you envision it? | - Positive experiences   Negative experiences  User friendly  Self-management/awareness  Exercise motivation  Motivation for protein intake  Role of physiotherapist and/or dietician  Aftercare |  |
| **Closing** | Is there anything else we haven't discussed yet that you think might still be important?  ✓ Summary  ✓ Thank interviewee  ✓ Explain next steps of project: results expected |  |  |
