# Supplementary material for: Promising patient experiences with a smartphone app and remote coaching for improving physical activity and protein intake to enhance recovery after oncological surgery: a multi-methods study
Source: Support Care Cancer. 2025 Jun 19;33(7):597. doi: 10.1007/s00520-025-09641-0 (PMC12178970; doi:10.1007/s00520-025-09641-0)
Supplement: Supplementary file 3 — Supplementary file3 (DOCX 15 KB) [file 520_2025_9641_MOESM3_ESM.docx]

## Appendix 3 - Phases of Thematic analysis described by Braun and Clark et al. (2023)

| **Phase Thematic analysis** | **Description** |
| --- | --- |
| 1. Familiarisation of the dataset | This phase involves reading and re-reading the data, to become immersed and intimately familiar with its content, and making notes on your initial analytic observations and insights, both in relation to each individual data item (e.g. an interview transcript) and in relation to the entire dataset. |
| 1. Generating codes | This phase involves generating succinct labels (codes) that capture and evoke important features of the data that might be relevant to addressing the research question. It involves coding the entire dataset, with two or more rounds of coding, and after that, collating all the codes and all relevant data extracts, together for later stages of analysis. |
| 1. Generating initial themes | This phase involves examining the codes and collated data to begin to develop significant broader patterns of meaning (potential themes). It then involves collating data relevant to each candidate theme, so that you can work with the data and review the viability of each candidate theme. |
| 1. Developing and reviewing themes | This phase involves checking the candidate themes against the coded data and the entire dataset, to determine that they tell a convincing story of the data, and one that addresses the research question. In this phase, themes are further developed, which sometimes involves them being split, combined, or discarded. In our Thematic Analysis approach, themes are defined as pattern of shared meaning underpinned by a central concept or idea (domains). |
| 1. Refining, defining and naming themes | This phase involves developing a detailed analysis of each theme, working out the scope and focus of each theme, determining the ‘story’ of each. It also involves deciding on an informative name for each theme. |
| 1. Writing the results | This final phase involves weaving together the analytic narrative and data extracts, and contextualizing the analysis in relation to existing literature. |
